# Clinical Specialty Expansion of AI-Enabled and Machine Learning–Enabled Medical Devices Authorized by the US Food and Drug Administration From 1995 to 2025: Longitudinal Content Analysis

**DOI:** 10.2196/103040

**Published:** 2026-08-25

**Authors:** Youn-Soo Lee, Bo-Young Youn

**Affiliations:** 1 Department of Digital Healthcare, College of Health and Medical Science, Daejeon University, #505 Moonmugwan 62, Daehak-ro, Dong-gu, Daejeon, Republic of Korea, +82-42-280-2062; 2 Department of Healthcare Management, College of Health and Medical Science, Daejeon University, #505 Moonmugwan 62, Daehak-ro, Dong-gu, Daejeon, 34520, Republic of Korea, +82-42-280-2062

**Keywords:** AI, machine learning, medical devices, US Food and Drug Administration, FDA authorization, clinical specialty, digital health, Software as a Medical Device, health informatics, innovation

## Abstract

**Background:**

The US Food and Drug Administration (FDA) has authorized AI-enabled and machine learning (ML)–enabled medical devices since 1995 and maintains a public registry of these authorizations. Prior analyses report that radiology dominates this landscape, but whether that concentration has persisted, intensified, or begun to reverse across 3 decades, particularly since 2022, remains insufficiently characterized.

**Objective:**

This study aimed to (1) characterize the longitudinal growth of FDA-authorized AI/ML-enabled devices from 1995 to 2025, (2) quantify the temporal evolution of clinical specialty distribution across 4 eras, (3) identify emerging specialties, and (4) examine the association between manufacturer type and nonradiology authorization.

**Methods:**

All 1430 devices in the FDA AI-Enabled Medical Devices registry (downloaded on March 1, 2026) with final marketing-authorization decisions through December 31, 2025, were analyzed. Devices were stratified by clinical specialty (FDA advisory committee panel) and 4 eras: Era 1 (1995‐2015), Era 2 (2016‐2019), Era 3 (2020‐2022), and Era 4 (2023‐2025). Concentration was quantified using the Herfindahl-Hirschman Index (HHI) with bootstrap CIs; the Cochran-Armitage test assessed trends in specialty share, with Bonferroni correction. Multivariable logistic regression estimated the odds of nonradiology authorization by manufacturer type and era, with an era-by-manufacturer interaction term. Sensitivity analyses used cluster-robust standard errors, a continuous authorization year variable, and Firth penalized regression. Manufacturers were classified using FDA records, Crunchbase, PitchBook, and company websites.

**Results:**

Annual authorizations rose from a mean of 2.0 (SD 2.0) in Era 1 to a mean of 264 (SD 58.2) in Era 4, with 331 authorizations in 2025 alone; the 510(k) pathway accounted for 96.2% (1376/1430). Radiology led in every era but followed a nonmonotonic trajectory, rising from 35.7% (15/42, Era 1) to a peak of 85.5% (347/406, Era 3) before declining to 77.5% (614/792, Era 4), the first significant decline on record (*P*=.001). The HHI fell from 0.738 (Era 3) to 0.612 (Era 4; bootstrap *P*<.001), indicating measurable diversification. Specialty distribution was associated with era (*χ*²_48_=328.0; *P*<.001; Cramér *V*=0.28). Compared with incumbents, start-ups (odds ratio [OR] 5.09, 95% CI 3.33‐7.79) and technology companies (OR 50.62, 95% CI 12.90‐198.64) had higher odds of nonradiology authorization; the nonsignificant era-by-manufacturer interaction (likelihood ratio test *χ*²_8_=11.16; *P*=.19) indicates a persistent rather than widening effect. The technology-company OR derives from only 13 devices across 5 firms; although directionally robust in sensitivity analyses, it is imprecise and warrants cautious interpretation.

**Conclusions:**

Radiology remained dominant, accounting for 77.5% (614/792) of Era 4 authorizations, but the specialty distribution showed measurable diversification during 2023 to 2025, associated with start-up and technology-company activity. Maturation of clinical data infrastructure beyond imaging is a plausible but unmeasured contributing condition, and authorization is not adoption. The findings bear on health-system readiness, workforce training, and specialty-specific regulatory frameworks.

## Introduction

### Background

AI and machine learning (ML) have emerged as transformative technologies in clinical medicine, enabling automated image interpretation, real-time patient monitoring, and data-driven clinical decision support across a widening range of medical specialties [1,2]. In the United States, the US Food and Drug Administration (FDA) serves as the primary regulatory authority for AI/ML-enabled medical devices and maintains a publicly accessible registry, the AI-Enabled Medical Devices List, that records authorizations spanning more than 3 decades [3]. This registry represents the most comprehensive longitudinal dataset on the regulatory authorization of clinical AI available in any country.

Despite the registry’s growing size, prior analyses have repeatedly identified a marked concentration of AI/ML device authorizations in radiology and related imaging disciplines [4,5]. Studies examining authorization cohorts through 2020 and 2024 have found that radiology has consistently accounted for approximately 75% to 80% of all authorized devices [4-6]. This concentration has been attributed to structural factors, including the early digitization of imaging data, the availability of large annotated training datasets, the well-defined task structure of image classification, and the dominance of established imaging-equipment manufacturers that integrated AI as a software layer on existing hardware platforms [7].

The clinical specialty in which an AI/ML device is authorized is not merely a descriptive attribute but a determinant of how, and by whom, these technologies are adopted at the bedside. Concentration in a single specialty implies that the benefits, risks, and infrastructure demands of clinical AI accrue unevenly across the health system, leaving specialties outside imaging with limited cumulative exposure to validation, workflow integration, and the AI literacy required for safe deployment [8]. A shift in this distribution would therefore signal a broader redistribution of these demands across the clinical workforce and would raise specialty-specific questions of postmarket monitoring and equity. As AI models extend into domains with less mature validation infrastructure, susceptibility to performance degradation and systematic bias against underrepresented patient subgroups may increase [9,10]. Characterizing whether, and where, the distribution is changing is thus a prerequisite for anticipating where these readiness demands will next arise.

Several converging developments since 2022 suggest that this concentration may be shifting. First, advances in deep learning and foundation models have catalyzed investment in AI applications across multiple clinical domains simultaneously, including cardiology, gastroenterology, pathology, and neurology [11]. Second, the FDA has expanded the product-code and advisory-committee classification system for AI devices, enabling authorization in specialties that previously lacked a clear regulatory pathway [3]. Third, the Predetermined Change Control Plan (PCCP) framework introduced in 2024 is intended to reduce regulatory friction for adaptive algorithms and may thereby facilitate authorization in specialties where AI is newer [6], although its actual effect on authorization patterns has not yet been evaluated empirically. Despite these developments, no study has systematically quantified whether specialty diversification across the full 3-decade authorization history is statistically significant or merely a marginal perturbation around a stable radiology-dominated baseline.

The distribution of authorizations across specialties is also shaped by the industrial structure of the manufacturers that pursue authorizations. Established imaging-equipment manufacturers have a strong incentive to develop AI as a complementary software layer that reinforces an existing hardware franchise, a dynamic that tends to concentrate incumbent activity within radiology. By contrast, the theory of disruptive innovation predicts that new entrants without an installed imaging base to defend will preferentially target clinical problems in which AI defines a novel workflow rather than augmenting an existing one [12,13]. If specialty diversification is indeed underway, the type of manufacturer driving it—whether a large incumbent, a small-to-mid-sized enterprise (SME), a start-up, or a technology company—becomes an informative marker of the underlying mechanism. Yet prior analyses of the FDA registry have characterized the device landscape predominantly by specialty and regulatory pathway and have rarely linked the observed specialty distribution to the structural characteristics of the firms securing authorization.

Beyond these substantive gaps, prior characterizations of the AI/ML device landscape have been methodologically constrained. Most have analyzed single-period cross sections, reporting the specialty composition of a cohort at one point in time rather than testing for change across the full authorization history [4-6]. Few have applied formal measures of market concentration or statistical tests for temporal trends, leaving open the question of whether year-to-year fluctuations in specialty share reflect a genuine directional shift or stochastic variation around a stable mean. A longitudinal design that spans the entire registry, quantifies concentration with an explicit index and confidence intervals, tests specialty trends across defined eras, and jointly models the contribution of manufacturer type is required to resolve this question and to situate the recent acceleration in authorizations within its proper historical context.

### Objectives

This study addressed 4 objectives. The first objective was to characterize the longitudinal growth trajectory of FDA-authorized AI/ML-enabled medical devices from 1995 to 2025, disaggregated by year and regulatory pathway. The second objective was to quantify temporal changes in the distribution of clinical specialties across 4 defined eras, using concentration indices and statistical tests for trend. The third objective was to identify specific clinical specialties exhibiting growth in authorization share during the recent acceleration period (2023‐2025). The fourth objective was to examine whether manufacturer type (incumbent, SME, start-up, or technology company) was associated with authorization in nonradiology specialties, and whether any such association changed over time. Nonradiology was defined as an analytic category comprising all specialties other than radiology; it therefore includes both long-established categories such as cardiovascular devices and newer, emerging categories such as pathology and gastroenterology-urology, and it is not synonymous with emerging specialties.

## Methods

### Data Source

This study used the FDA AI-Enabled Medical Devices List as the primary data source [3]. This registry is maintained by the FDA Center for Devices and Radiological Health and includes devices identified primarily through the presence of AI-related terms in the summary descriptions of marketing-authorization documents or through device classification codes. The FDA updates this registry on an ongoing basis as new authorizations are added; a fixed cutoff date was therefore applied. The complete registry was downloaded on March 1, 2026, and the analysis was restricted to devices with a date of final decision on or before December 31, 2025; devices with final decisions after this cutoff date were excluded. The final analytic dataset consisted of 1430 devices, with final decision dates ranging from September 29, 1995, to December 30, 2025. The study period from 1995 to 2025 therefore refers to the dates of final marketing-authorization decisions rather than to the date of registry access. For each device, the following fields were extracted: date of final decision, submission number, device name, company name, advisory committee panel (clinical specialty), and primary product code. Submission numbers were used to retrieve decision summaries from the corresponding regulatory databases (510(k), Premarket Approval [PMA], and De Novo) to supplement specialty and manufacturer information as needed. The unit of analysis was the individual marketing authorization: each row in the registry corresponds to a unique submission number, and no duplicate submission numbers were present. All 1430 records meeting the cutoff date were included, with no further exclusion criteria applied. Updated versions of a previously authorized device that received a new marketing authorization under a separate submission number were retained as distinct records, in keeping with the focus of this study on authorization activity rather than on the number of unique products.

The present data source differs fundamentally from the source used by Yu et al [7], who applied keyword filtering to the broader openFDA Software as a Medical Device (SaMD) database to identify SaMD products. The present analysis uses a dedicated AI/ML-specific registry that identifies devices based on AI/ML technological characteristics. This distinction is material: the earlier dataset captured all SaMD (including non-AI software), whereas the present dataset captures AI/ML devices specifically (including both SaMD and devices with AI/ML features embedded in hardware-software combination products).

### Era Classification

Authorizations were classified into 4 eras based on the trajectory of authorization volume and key technological milestones. Era 1 (September 1995-December 2015) was the early period, characterized by very low annual authorization volume (a mean of 2.0, SD 2.0, devices per year) and a heterogeneous specialty mix. Era 2 (January 2016-December 2019) was the growth period, coinciding with the deep-learning revolution and the first dedicated FDA AI/ML guidance activity. Era 3 (January 2020-December 2022) was the acceleration period, coinciding with the COVID-19-driven expansion of digital-health investment. Era 4 (January 2023-December 2025) was the maturation period, characterized by foundation model development, the introduction of the PCCP framework, and the highest authorization volume on record. The era boundaries were specified a priori on the basis of these volume and milestone considerations rather than to equalize interval length; the 4 eras therefore differ in duration (approximately 21, 4, 3, and 3 years, respectively).

Given that direct comparisons of raw counts across eras of unequal length can be misleading, era-level comparisons were expressed as within-era proportions or mean annual rates, and a sensitivity analysis replacing the era categories with authorization year as a continuous covariate was performed on the logistic regression model (see “Statistical Analysis” section). Specifically, 2016 was selected as the first cut point, marking the arrival of deep-learning-based devices and the first dedicated FDA AI/ML guidance activity; 2020 was selected for the abrupt expansion of digital-health investment and remote-care deployment that followed the onset of the COVID-19 pandemic; and 2023 was selected for the emergence of foundation models, the development of the PCCP framework, and a step change resulting in the highest annual authorization volumes on record.

### Specialty Classification

Clinical specialty was classified using the FDA advisory committee panel designation included in the registry. The FDA uses advisory committee panels corresponding to medical specialties (eg, Radiology, Cardiovascular, Neurology, Gastroenterology-Urology, Pathology, Ophthalmic, Hematology, Obstetrics and Gynecology, Anesthesiology, and General and Plastic Surgery). When an advisory committee designation was absent, the specialty was assigned based on the primary product code, using the FDA Product Code Classification Database. Each device was assigned to a single primary specialty. Accordingly, throughout this article, the term specialty denotes the FDA advisory committee panel designation, which is a regulatory classification and does not necessarily correspond to the clinical setting in which a device is ultimately deployed. In particular, a device authorized under one panel may be used in multidisciplinary clinical workflows; for example, an imaging-based triage tool classified under the Radiology panel may be acted on primarily by emergency physicians or neurologists. In an exploratory analysis, devices classified under the Radiology panel were further subclassified by imaging modality (computed tomography [CT], magnetic resonance imaging [MRI], ultrasound, X-ray or fluoroscopy, nuclear medicine, mammography, dental imaging, or radiation therapy planning), with the modality identifiable from the device name and product code.

### Manufacturer Classification

For each unique company in the dataset, manufacturers were classified into 1 of 4 mutually exclusive types by applying the following criteria hierarchically, with each company assigned to the first category whose criteria it met: (1) technology company (an established software or consumer-technology firm diversifying into medical AI, irrespective of size), (2) large incumbent (more than 1000 employees and established before 2000), (3) start-up (founded in 2010 or later and preinitial public offering or early stage), and (4) SME (11-999 employees), the residual category comprising established small-to-mid-sized firms founded before 2010 and formerly early-stage firms that had matured to a public listing or acquisition. Under this hierarchy, a company founded in 2010 or later with, for example, 200 employees was classified as a start-up while it remained preinitial public offering or early stage, and as an SME otherwise; the start-up and SME categories, therefore, could not overlap. Manufacturer profiles were retrieved from FDA records, Crunchbase, PitchBook, and official company websites. Classification of the highest-volume manufacturers was verified individually; the remaining, lower-volume manufacturers were classified based on founding year and naming conventions, a limitation acknowledged below. Classification criteria and the resulting category counts are provided in Table S6 of Multimedia Appendix 1.

### Statistical Analysis

Descriptive statistics (counts, proportions, and time series) characterized the dataset. The Herfindahl-Hirschman Index (HHI), defined as the sum of squared specialty share fractions, was calculated for each era to quantify specialty concentration (range: 0-1; higher values indicate greater concentration), with 95% CIs obtained from 2000 bootstrap resamples. The change in HHI from Era 3 to Era 4 was assessed by drawing 2000 independent bootstrap resamples of the device sets within each of the 2 eras, computing the difference in HHI for each pair of resamples, and calculating the 2-sided *P* value as twice the proportion of resampled differences less than or equal to zero. The Cochran-Armitage test assessed linear trends in the proportion of authorizations by specialty across eras, and a 2-proportion *z*-test compared the radiology share between Era 3 and Era 4. For the trend tests, the 4 eras were coded as equally spaced ordinal scores (1-4) in chronological order; the test therefore evaluates ordered change in specialty share across successive eras rather than change per calendar year, so the unequal era durations do not enter the test statistic.

The association between specialty and era was tested with the Pearson chi-square test, and the effect size was reported as the Cramér *V* statistic. In the 17-panel by 4-era contingency table, 51 of the 68 cells had expected counts below 5, violating the conventional rule of thumb for the asymptotic chi-square approximation; the result was therefore verified with a Monte Carlo permutation test (10,000 replicates), which yielded the same conclusion (Table S8, Multimedia Appendix 1). A multivariable logistic regression model was specified with binary specialty classification (nonradiology vs radiology) as the outcome and era and manufacturer type as predictors (reference categories: Era 1 and large incumbent). An era-by-manufacturer interaction term was added to a second model, and the interaction’s contribution was assessed using a likelihood ratio test. Multiple devices may originate from the same manufacturer, creating within-manufacturer correlation that can lead model-based standard errors to be underestimated; as a sensitivity analysis, the main-effects model was therefore reestimated with cluster-robust standard errors clustered at the manufacturer level (576 clusters). Two additional sensitivity analyses were performed: a model replacing the era categories with authorization year as a continuous covariate to confirm that the estimates were not artifacts of the unequal era durations, and a Firth penalized logistic regression to address potential small-sample bias arising from the small technology-company category (5 manufacturers, 13 devices); full results of all sensitivity analyses are provided in Table S7 of Multimedia Appendix 1. Cochran-Armitage trend tests were performed for all 17 advisory committee panels, and the resulting family of 17 *P* values was Bonferroni-corrected. Uncorrected *P* values for the 10 nonradiology specialties are reported in Table S7 of Multimedia Appendix 1; *Z* statistics with both uncorrected and Bonferroni-adjusted *P* values for all 17 panels are provided in Table S4 of Multimedia Appendix 1. Wherever a trend is described as statistically significant in the text, the Bonferroni-adjusted *P* value is used, and any trend reaching significance only before correction is identified explicitly as such. Per-era HHI values with bootstrap CIs are tabulated in Table S5 of Multimedia Appendix 1. Statistical significance was set at *P*<.05. Analyses were performed in Python (version 3.9; Python Software Foundation) using the pandas, SciPy, and statsmodels libraries.

### Ethical Considerations

This study was a secondary analysis of publicly available regulatory records from the FDA AI-Enabled Medical Devices List and the associated FDA device databases, which describe medical devices and their manufacturers. The study did not involve human participants, and no patient data or personally identifiable information was collected, accessed, or analyzed. As the analyzed databases contained no patient-identifiable information, ethics review board approval and informed consent were not required, in accordance with the US Federal Policy for the Protection of Human Subjects (45 CFR §46.102), under which research that does not obtain information about living individuals does not constitute human subjects research; accordingly, no application for an ethics review board assessment was submitted.

## Results

### Overview: 30 Years of Authorization Growth

**Figure 1.**
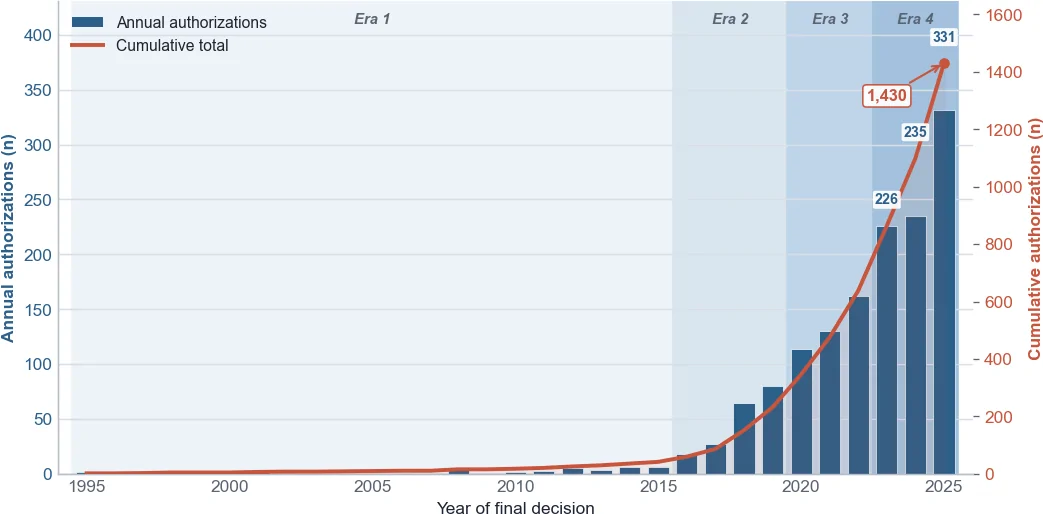
Annual and cumulative (30-year) authorizations of AI-enabled and machine learning (ML)–enabled medical devices by the US Food and Drug Administration (FDA), 1995‐2025 (N= 1430). Shaded bands denote the 4 eras. The registry was downloaded on March 1, 2026, and restricted to devices with final decisions on or before December 31, 2025; the 2025 count is, therefore, a complete calendar-year total.

Between September 1995 and December 2025, the FDA authorized 1430 AI/ML-enabled medical devices, spanning 576 unique manufacturers and 17 advisory committee specialty panels. Era 1 (1995‐2015) contributed 42 devices (2.9%), Era 2 (2016‐2019) contributed 190 devices (13.3%), Era 3 (2020‐2022) contributed 406 devices (28.4%), and Era 4 (2023‐2025) contributed 792 devices (55.4%), for a total of 1430 devices. Annual authorization volume increased from a mean of 2.0 devices per year in Era 1 to 264 devices per year in Era 4, with 331 authorizations recorded in 2025 alone (Figure 1). The 2025 count reflects the prespecified cutoff date of December 31, 2025 (registry downloaded on March 1, 2026) and is therefore a complete calendar-year total. The 510(k)-pathway accounted for the majority of authorizations (1376 devices, 96.2%), followed by the De Novo pathway (37 devices, 2.6%) and the PMA pathway (17 devices, 1.2%).

### Temporal Evolution of Clinical Specialty Distribution

**Figure 2.**
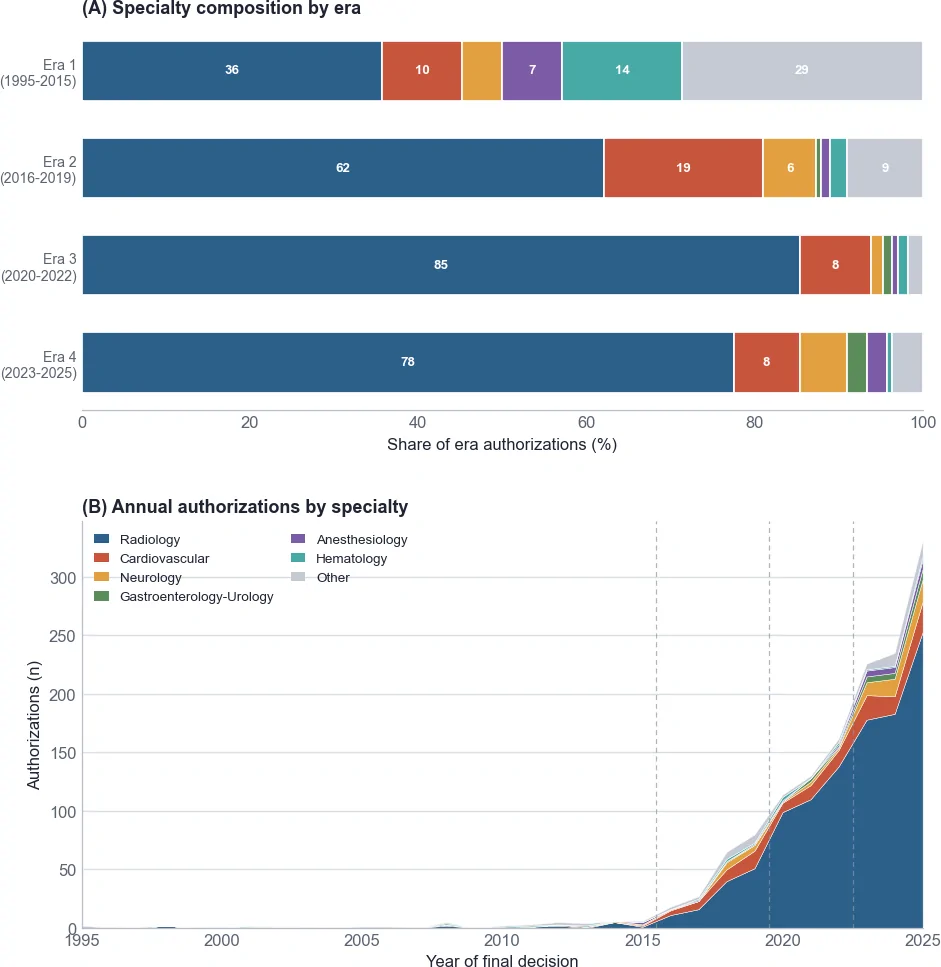
Clinical specialty distribution of FDA AI/ML–enabled medical devices over time, shown as (A) proportions and (B) counts. Era boundaries are marked by vertical dotted lines. FDA: US Food and Drug Administration; ML: machine learning.

Radiology was the leading specialty in all 4 eras, but the radiology share followed a nonmonotonic trajectory rather than a simple monotonic decline (Table 1; Figure 2). The radiology share rose from 35.7% (95% CI 23.0%‐50.8%) in Era 1 to 62.1% (95% CI 55.0%‐68.7%) in Era 2, peaked at 85.5% (95% CI 81.7%‐88.6%) in Era 3, and then declined to 77.5% (95% CI 74.5%‐80.3%) in Era 4. An Era 3-to-Era 4 decline of 7.9 percentage points was statistically significant (2-proportion *z* test; *P*=.001) and represents the first significant reduction in radiology share on record.

**Table 1.** Distribution of authorizations by clinical specialty and era, with the Herfindahl-Hirschman Index (HHI) per era^a^.

Specialty	Era 1 (1995‐2015), n (%)	Era 2 (2016‐2019), n (%)	Era 3 (2020‐2022), n (%)	Era 4 (2023‐2025), n (%)
Radiology	15 (35.7)	118 (62.1)	347 (85.5)	614 (77.5)
Cardiovascular	4 (9.5)	36 (18.9)	34 (8.4)	62 (7.8)
Neurology	2 (4.8)	12 (6.3)	6 (1.5)	45 (5.7)
Anesthesiology	3 (7.1)	2 (1.1)	3 (0.7)	18 (2.3)
Gastroenterology-urology	0 (0)	1 (0.5)	4 (1.0)	19 (2.4)
Hematology	6 (14.3)	4 (2.1)	5 (1.2)	5 (0.6)
Ophthalmic	0 (0)	2 (1.1)	4 (1.0)	4 (0.5)
Pathology	2 (4.8)	0 (0)	1 (0.2)	6 (0.8)
Other (n=9 specialties)	10 (23.8)	15 (7.9)	2 (0.5)	19 (2.4)
Total devices (n)	42	190	406	792
Active specialties (n)	11	15	10	15
HHI	0.181	0.427	0.738	0.612

a Values are n (% of era total). Other comprises the 9 specialties with the fewest authorizations. Percentages are column proportions within each era. The complete distribution across all 17 advisory committee panels is provided in Table S2 of Multimedia Appendix 1.

**Figure 3.**
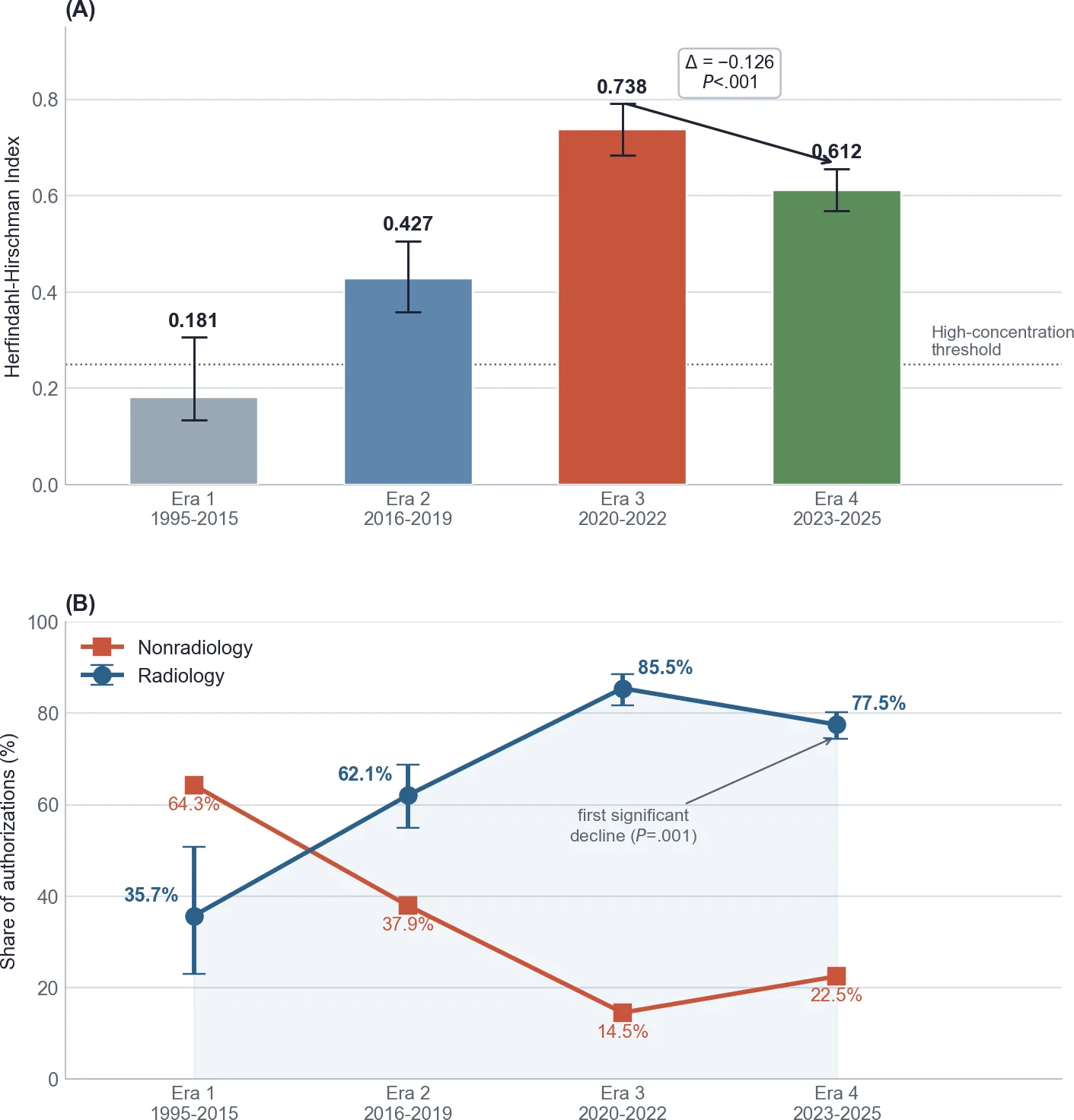
Concentration dynamics across eras. (A) Herfindahl-Hirschman Index with bootstrap 95% CIs. (B) Radiology vs nonradiology shares with Wilson 95% CIs.

The HHI tracked this trajectory, increasing from 0.181 (95% CI 0.134‐0.306) in Era 1 to a maximum of 0.738 (95% CI 0.684‐0.791) in Era 3, and then decreasing to 0.612 (95% CI 0.569‐0.656) in Era 4 (Figure 3). The reduction in HHI from Era 3 to Era 4 was statistically significant (bootstrap *P*<.001), indicating measurable specialty diversification during the most recent era. The association between specialty distribution and era was significant overall (*χ*²_₄₈_=328.0; *P*<.001; Cramér *V*=0.28); given the sparse cells in the full 17-panel-by-4-era table, this association was verified with a Monte Carlo permutation test (10,000 replicates; *P*<.001; Table S8, Multimedia Appendix 1); the complete distribution across all 17 advisory committee panels is provided in Table S2 of Multimedia Appendix 1.

### Imaging Modalities Within Radiology

In an exploratory analysis of 1094 radiology devices, imaging modality was identifiable from the device name or product code for 629 (57.5%). Among these 629 devices with an identifiable modality, CT was the most common (n=200), followed by ultrasound (n=124), MRI (n=109), x-ray or fluoroscopy (n=51), mammography (n=44), dental imaging (n=41), radiation therapy planning (n=34), and nuclear medicine (n=26). The remaining 465 radiology devices (42.5%) were general image-processing or image-management software for which a single modality could not be assigned from the available fields. Because a modality could not be assigned to 42.5% of radiology devices, these modality findings are exploratory and pertain only to the identifiable subset. The full modality distribution by era is provided in Table S1 of Multimedia Appendix 1.

### Emerging Specialties in Era 4

**Figure 4.**
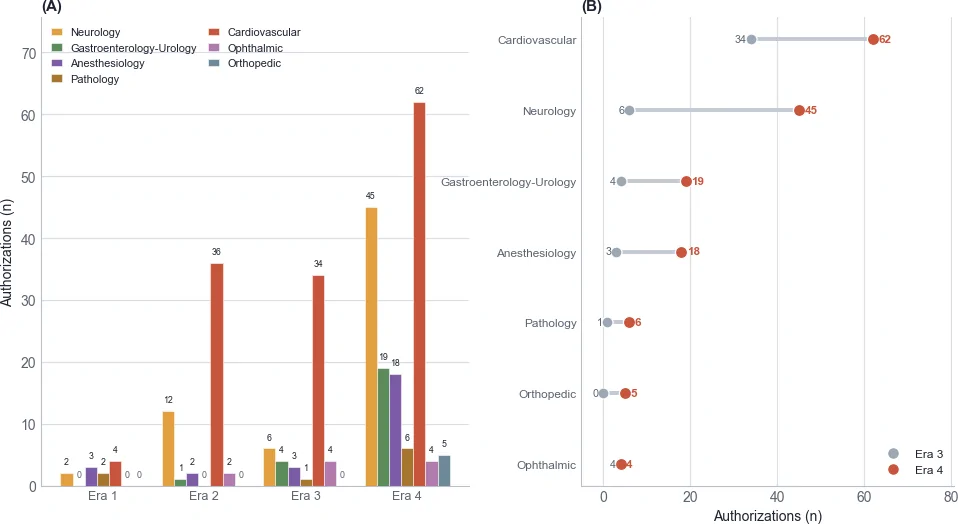
Emerging specialties in Era 4 (2023-2025). (A) Nonradiology specialty counts by era. (B) Change in authorization counts from Era 3 to Era 4 for each nonradiology specialty, with exact counts shown. As the eras are of equal length (3 years each), the change in counts is proportional to the change in mean annual authorization rate. Era 3-to-Era 4 rate ratios are unstable for specialties with small Era 3 counts (eg, pathology, with 1 Era 3 authorization) and should be interpreted with caution

In this study, emerging specialties were defined descriptively as nonradiology specialties showing pronounced growth in mean annual authorization rate between Era 3 and Era 4, not as specialties exhibiting a statistically significant linear trend across all 4 eras; accordingly, some specialties with large Era 4 increases (eg, neurology: *Z*=+0.84; Bonferroni-adjusted *P*>.99) did not show significant Cochran-Armitage trends over the full study period. These 2 sets of statistics are complementary rather than contradictory: the Cochran-Armitage statistic tests for a monotonic trend in specialty share across all 4 eras, so a specialty whose share was low and approximately flat through Era 3 and then rose only in Era 4, such as neurology, anesthesiology, or pathology, can show a large Era 4 to Era 3 rate ratio without a significant overall trend. The pattern for these specialties is, therefore, best described as a recent, era-specific increase rather than a consistent trend across the entire study period. Among nonradiology specialties, neurology, gastroenterology-urology, pathology, and anesthesiology demonstrated the most pronounced growth between Era 3 and Era 4 on an annual-rate basis (Figure 4; Table 2). Neurology authorizations increased from 6 (Era 3) to 45 (Era 4), a 7.5-fold increase in annual rate; gastroenterology-urology increased from 4 to 19 (4.8-fold); anesthesiology increased from 3 to 18 (6.0-fold); and pathology increased from 1 to 6 (6.0-fold). Orthopedic devices first appeared in Era 4 (5 devices). Cardiovascular devices, the largest nonradiology category overall (136 devices), increased in absolute terms from 34 (Era 3) to 62 (Era 4) but declined in relative share across the full period (Cochran-Armitage *Z*=−3.44; Bonferroni-adjusted *P*=.010). None of the 4 specialties identified above as emerging showed a statistically significant Cochran-Armitage trend after Bonferroni correction (neurology: *Z*=+0.84, Bonferroni-adjusted *P*>.99; gastroenterology-urology: *Z*=+2.34, Bonferroni-adjusted *P*=.33; anesthesiology: *Z*=+0.14, Bonferroni-adjusted *P*>.99; pathology: *Z*=−0.51, Bonferroni-adjusted *P*>.99). Gastroenterology-urology was the only one to reach significance before correction (*Z*=+2.34; uncorrected *P*=.02; Bonferroni-adjusted *P*=.33), and its growth is, therefore, reported here as an era-specific increase rather than as a significant trend across the study period. Apart from the increasing radiology trend (*Z*=+5.30; Bonferroni-adjusted *P*<.001), the only trends that remained significant after correction were declines in the relative share of cardiovascular (*Z*=−3.44; Bonferroni-adjusted *P*=.010), hematology (*Z*=−5.01; Bonferroni-adjusted *P*<.001), clinical chemistry (*Z*=−3.37; Bonferroni-adjusted *P*=.013), microbiology (*Z*=−3.58; Bonferroni-adjusted *P*=.006), and clinical toxicology (*Z*=−5.90; Bonferroni-adjusted *P*<.001) devices, each of which was concentrated in the earlier eras (Table S4, Multimedia Appendix 1). The number of advisory committee panels receiving at least one authorization rose from 10 in Era 3 to 15 in Era 4.

**Table 2.** Nonradiology specialties by era, with the Era 4-to-Era 3 annual-rate ratio and the Cochran-Armitage trend statistic (*Z*) across all 4 eras^a^.

Specialty	Era 1, n	Era 2, n	Era 3, n	Era 4, n	Total, N	Era 4 share, %	Era 3 annual mean (SD)	Era 4 annual mean (SD)	Era 4 or Era 3 rate ratio	*Z*	*P* value^b^
Cardiovascular	4	36	34	62	136	7.8	11.3 (3.1)	20.7 (5.5)	1.8×	−3.44	<.001
Neurology	2	12	6	45	65	5.7	2.0 (1.0)	15.0 (4.0)	7.5×	0.84	.40
Anesthesiology	3	2	3	18	26	2.3	1.0 (1.0)	6.0 (1.7)	6.0×	0.14	.89
Gastroenterology-urology	0	1	4	19	24	2.4	1.3 (1.5)	6.3 (2.3)	4.8×	2.34	.02
Hematology	6	4	5	5	20	0.6	1.7 (1.5)	1.7 (1.2)	1.0×	−5.01	<.001
Ophthalmic	0	2	4	4	10	0.5	1.3 (0.6)	1.3 (0.6)	1.0×	−0.63	.53
Pathology	2	0	1	6	9	0.8	0.3 (0.6)	2.0 (1.7)	6.0×	−0.51	.61
Orthopedic	0	0	0	5	5	0.6	0 (0)	1.7 (1.2)	—^c^	1.74	.08
Dental	0	2	0	3	5	0.4	0 (0)	1.0 (1.7)	—^c^	−0.44	.66
Obstetrics and gynecology	1	1	0	2	4	0.3	0 (0)	0.7 (1.2)	—^c^	−1.49	.14

a Era 1 through Era 4: authorization counts per era. Era 4 or Era 3 rate ratio: ratio of mean annual authorization rates between the 2 eras. *Z*: Cochran-Armitage trend test statistic across all 4 eras; a negative value indicates a declining share. *P* values are uncorrected for multiple comparisons. Bonferroni-adjusted *P* values (correcting for the 17 advisory committee panels tested) are provided in Table S4 of Multimedia Appendix 1. No nonradiology specialty listed in this table showed a significant increasing trend after Bonferroni correction. Era 3 and Era 4 annual means are the mean authorizations per year (era counts divided by 3).

b *P* values are uncorrected.

c Rate ratio was not calculable, as no authorizations occurred in Era 3; orthopedic devices were newly observed in Era 4, whereas dental devices and obstetrics and gynecology devices had authorizations in earlier eras but none in Era 3.

### Manufacturer Type and Specialty Expansion

Among 576 unique manufacturers, 70 were classified as large incumbents (385/1430, 26.9% devices), 27 as SMEs (104/1430, 7.3% devices), 474 as start-ups (928/1430, 64.9% devices), and 5 as technology companies (13/1430, 0.9% devices). The radiology share differed markedly by manufacturer type: 93.0% (358/385) for large incumbents, 71.8% (666/928) for start-ups, 64.4% (67/104) for SMEs, and 23.1% (3/13) for technology companies.

**Figure 5.**
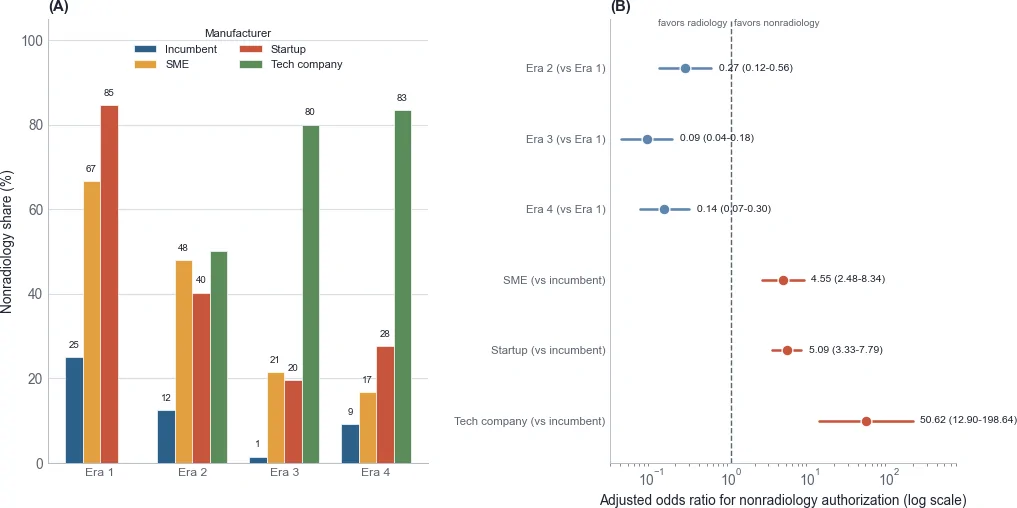
Manufacturer type, era, and specialty expansion. (A) Nonradiology authorization share by manufacturer type and era. (B) Multivariable logistic regression odds ratios for nonradiology authorization (reference: Era 1, incumbent). SME: small-to-mid-sized enterprise.

In the multivariable logistic regression model, both start-ups (odds ratio [OR] 5.09, 95% CI 3.33‐7.79; *P*<.001) and technology companies (OR 50.62, 95% CI 12.90‐198.64; *P*<.001; an estimate derived from only 13 devices authorized by 5 firms and therefore highly imprecise) showed substantially higher odds of nonradiology authorization compared with incumbents, after adjustment for era (Figure 5). SMEs also showed higher odds (OR 4.55, 95% CI 2.48‐8.34; *P*<.001). The addition of an era-by-manufacturer interaction term did not significantly improve model fit (likelihood ratio test *χ*²_8_=11.16; *P*=.19), indicating that the manufacturer association with nonradiology authorization was persistent across eras rather than widening over time. Full coefficients for the era-by-manufacturer interaction model are provided in Table S3 of Multimedia Appendix 1. The manufacturer associations were robust across sensitivity analyses (Table S7, Multimedia Appendix 1). With cluster-robust SEs at the manufacturer level (576 clusters), the point estimates were unchanged and remained statistically significant despite wider CIs (start-up: OR 5.09, 95% CI 2.04‐12.71; SME: OR 4.55, 95% CI 1.24‐16.68; technology company: OR 50.62, 95% CI 7.92‐323.56). Replacing the era categories with authorization year as a continuous covariate yielded similar estimates (start-up: OR 5.37; SME: OR 5.24; technology company: OR 45.28), as did Firth-penalized logistic regression (start-up: OR 4.99, 95% CI 3.27‐7.61; SME: OR 4.50, 95% CI 2.46‐8.21; technology company: OR 44.44, 95% CI 11.71‐168.65). These estimates should nevertheless be interpreted in light of residual manufacturer-classification uncertainty, the repeated contribution of devices from the same manufacturers (addressed through the cluster-robust standard errors), and, in particular, the small technology-company category (5 manufacturers, 13 devices), whose estimate should be interpreted cautiously despite its robustness in the sensitivity analyses. For the technology-company category specifically, the sensitivity analyses establish that the direction and approximate order of magnitude of the association are robust, but the confidence intervals span more than an order of magnitude in every specification; the point estimate of 50.62 should therefore be read as evidence of a strong association rather than as a precise quantification of its size.

## Discussion

### Principal Findings

This longitudinal analysis of 1430 FDA-authorized AI/ML medical devices characterizes the temporal evolution of clinical specialty distribution in medical AI across the full 3-decade span of the FDA registry, from 1995 to 2025. Three principal findings emerge. First, the dominance of radiology is persistent but no longer monotonically increasing: the radiology share rose through Era 3, peaked at 85.5% (347/406), and then declined to 77.5% (614/792) in Era 4, the first statistically significant reduction on record, a reversal corroborated by a significant decrease in the HHI. The structural advantages that established this dominance remain influential but appear to be diminishing in relative influence. The early digitization of imaging through the Digital Imaging and Communications in Medicine standard introduced in the 1980s and 1990s created a structured, machine-readable data substrate decades before equivalent infrastructure existed in other specialties [14,15]. That head start, combined with the well-defined task structure of image interpretation and the integration of AI as a software layer by established imaging-equipment manufacturers, allowed deep-learning methods to mature first in medical imaging [16]. The continued breadth of radiology applications, spanning lesion detection, triage, quantification, and image reconstruction, sustains a high baseline of radiology authorizations even as other specialties grow [17]. Second, diversification during Era 4 is concentrated in a defined set of emerging domains, including neurology, gastroenterology-urology, pathology, and anesthesiology. These domains were identified descriptively, from their Era 3-to-Era 4 growth in mean annual authorization rate; the enabling conditions discussed below are proposed explanations rather than factors measured in this study. The growth of these domains is recent and era-specific rather than continuous across the full study period, which is why their Cochran-Armitage trend statistics are nonsignificant after Bonferroni correction (all Bonferroni-adjusted *P*≥.33) despite large Era 4 rate increases. Third, start-ups and technology companies are disproportionately associated with nonradiology authorizations, and this association is persistent across eras rather than a recent phenomenon. The start-up association is estimated from 928 authorizations and is correspondingly precise, whereas the technology-company association is estimated from 13 authorizations by 5 firms; the latter is directionally consistent across every sensitivity analysis but is too imprecise to support a claim about the magnitude of the difference. At the same time, the magnitude of this diversification should not be overstated: radiology still accounted for 77.5% (614/792) of Era 4 authorizations, and the observed shift represents redistribution at the margin rather than a displacement of radiology’s central position in medical AI.

### Factors Potentially Underlying Specialty Expansion

Three factors may help explain the observed specialty expansion; because this study is a content analysis of authorization records, these factors should be understood as plausible explanations rather than demonstrated causal mechanisms. The first factor is data-infrastructure maturation. The growth in pathology authorizations follows the digitization of pathology departments and the assembly of large, annotated whole-slide imaging datasets, which have enabled clinical-grade deep learning applications in histopathology [18,19]. Weakly supervised learning on whole-slide images has reduced the dense-annotation burden that previously constrained computational pathology, shortening the path from research prototype to authorized product [20]. The growth in gastroenterology authorizations follows investment in annotated endoscopy video datasets for colonoscopy polyp detection [21], an application subsequently validated in randomized trials that demonstrated improved adenoma detection rates [22]. This pattern extends the data-accessibility thesis articulated by Yu et al [7]: as structured data accumulate in a clinical specialty, the conditions for AI authorization become favorable.

The second factor is clinical demand combined with established performance benchmarks. Cardiovascular disease remains a leading cause of death globally, and electrocardiogram (ECG) interpretation, echocardiographic analysis, and cardiac-rhythm monitoring represent high-volume clinical tasks with well-defined reference standards. Deep-learning algorithms for ECG interpretation have achieved cardiologist-level performance in benchmark studies [23] and have demonstrated the capacity to detect conditions not readily apparent to human readers, such as paroxysmal atrial fibrillation inferred from a normal sinus-rhythm tracing [24]. The accumulation of large labeled ECG archives and the integration of AI into established cardiac-monitoring workflows have positioned cardiovascular medicine as the largest nonradiology category in the present dataset [25]. This describes the absolute standing of cardiovascular medicine rather than its trajectory: although cardiovascular authorizations rose from 34 in Era 3 to 62 in Era 4, the cardiovascular share of all authorizations declined significantly across the 4 eras (Bonferroni-adjusted *P*=.010), because radiology and, more recently, other nonradiology specialties grew faster. The maturation of large language models and multimodal foundation models has further broadened the range of clinical tasks amenable to automation beyond image classification, extending into clinical text, structured records, and multimodal reasoning [26,27].

The third factor is regulatory-pathway evolution. The De Novo pathway has been used for AI devices in novel specialties that lack a suitable predicate, and the PCCP framework introduced in 2024 is designed to reduce regulatory friction for adaptive algorithms whose performance is expected to improve with deployment data [6]; however, this study did not directly analyze PCCP use, and its contribution to specialty diversification remains a plausible hypothesis rather than an established effect. Moreover, given that the PCCP framework was introduced only in 2024, near the end of the study period, its influence on the Era 4 trends observed here is likely to have been limited. The need for a system-level regulatory view of continuously learning algorithms has been articulated repeatedly [28], and the FDA action plan on continual learning represents an early response to that need [29]. The progressive lowering of data barriers outside radiology, supported by federated-learning platforms that enable multi-institutional model training without sharing patient data [30] and by standardized pipelines for preparing medical-imaging data for ML [31], has reduced the historical constraints on AI development in nonimaging specialties, although privacy and governance challenges remain substantial [32].

### Manufacturer Dynamics and Innovation

The finding that start-ups and technology companies are disproportionately associated with nonradiology authorizations is consistent with, although it does not by itself demonstrate, established theory on disruptive innovation in health care [12,13]. A plausible interpretation is that incumbent imaging-equipment manufacturers develop AI predominantly as a complementary product that strengthens an existing imaging-hardware franchise, which would concentrate incumbent activity in radiology (358/385, 93.0% radiology share), whereas start-ups and technology companies, lacking an installed imaging base to defend, may pursue applications in specialties where AI defines a new clinical workflow rather than augmenting an existing one. As this study did not directly examine company strategy, these explanations are interpretations consistent with the observed authorization patterns rather than demonstrated corporate behavior. The persistence of this association across eras (nonsignificant era-by-manufacturer interaction) suggests that the structural division of innovative labor between incumbents and new entrants is a durable feature of the medical-AI industry rather than a recent development. The diversification observed in Era 4 may therefore reflect less a change in start-up behavior than the substantial increase in the number of start-up entrants, with start-ups accounting for 928 of 1430 (64.9%) authorizations. Technology-company authorizations were also the least concentrated in radiology of any manufacturer type (3/13, 23.1% radiology share), consistent with the consumer-device origins of these firms and the large volumes of physiological-signal data generated by consumer hardware platforms; with only 13 such authorizations observed, however, their distribution across individual specialties was not analyzed, and no inference about specific application areas is drawn here.

### Implications for Health Systems and Policy

The expansion of specialty AI/ML medical devices, although still modest relative to radiology’s share of authorizations, carries implications for health system readiness. As AI moves beyond the radiology reading room into procedural and primary care specialties, workforce training and clinical integration models must adapt accordingly [8]. Radiology has had approximately a decade to develop AI literacy and workflow integration; cardiology, gastroenterology, and pathology are earlier in the process. Operationally, specialty expansion implies that each newly affected field will need to build the capabilities that radiology has accumulated over the past decade: specialty-specific AI literacy among the clinicians who act on model outputs; local validation of models on institutional data before deployment; ongoing performance monitoring to detect degradation or drift after deployment; deliberate integration of model outputs into specialty-specific clinical workflows; and governance structures that assign responsibility for the procurement, oversight, and decommissioning of clinical AI. Successful clinical translation will require sustained attention to validation, monitoring, and integration challenges that extend well beyond regulatory authorization [9]. Algorithmic bias is a particular concern as AI extends into specialties with less mature validation infrastructure because models trained on nonrepresentative data can systematically disadvantage patient subgroups and entrench existing inequities [10]. The availability of the PCCP framework for recent authorizations also creates a need for robust postmarket surveillance, particularly in specialties where real-world performance may diverge from premarket study conditions [9]. Medical-education curricula and continuing professional development programs will accordingly need to incorporate specialty-specific AI competencies so that clinicians outside radiology can appraise, deploy, and monitor these tools responsibly [33].

### Comparison With Prior Literature

The present findings are consistent with, and extend, prior analyses. Benjamens et al [4] reported radiology’s dominance through 2020, and an analysis of 2024 authorizations reported continued dominance at approximately 75% to 80%, with growth in pathology and cardiovascular devices [6]. Those studies characterized single-period cohorts; the present longitudinal analysis across 3 decades provides statistical confirmation that the shift is significant and era-dependent rather than anecdotal, and identifies Era 4 as the first period in which radiology’s share fell significantly. Relative to the SaMD innovation study of Yu et al [7], this study is complementary but distinct: where the earlier work analyzed SaMD broadly (AI and non-AI) using openFDA keyword filtering and ended in 2022, this study analyzes AI/ML devices specifically using the dedicated FDA AI registry and extends 3 additional years through the highest-volume authorization era on record. The comparative regulatory analysis of Muehlematter et al [5] documented similar concentration patterns in the United States and Europe through 2020; whether the diversification observed here also appears under the European Union Medical Device Regulation framework is an open question for future cross-jurisdictional study.

### Limitations

Several limitations should be acknowledged. First, the FDA AI-Enabled Medical Devices List is not exhaustive: the registry reflects devices identified primarily through AI-related terms in decision summaries, and devices may be missed if AI/ML features are not prominently described in regulatory filings, a constraint that compounds known limitations in how AI device performance is reported [34]. Second, the advisory committee panel designation reflects regulatory classification rather than clinical deployment context, so a device classified under Radiology may ultimately be used in a multidisciplinary setting, and imaging modality could be determined for only 57.5% (629/1094) of radiology devices from the available fields. Third, classification of lower-volume manufacturers as start-ups versus SMEs relied on founding year and naming conventions for the large number of single-device companies, and misclassification may affect logistic regression estimates; the magnitude and direction of the principal manufacturer effect, however, were large and robust. In addition, the technology-company category comprised only 5 manufacturers and 13 devices; although the association persisted under Firth penalized estimation and manufacturer-level cluster-robust standard errors, the wide CI warrants cautious interpretation of this estimate. Fourth, the analysis covers only the United States, and the European Union, Japan, South Korea, and China have distinct regulatory pathways that may yield different specialty distribution patterns. Fifth, FDA authorization does not necessarily indicate clinical adoption, reimbursement, routine use, or real-world impact; this study characterizes regulatory authorization patterns, not clinical implementation. Sixth, as the unit of analysis was the individual authorization, multiple authorizations from the same manufacturer or product family can inflate device counts and induce correlation among observations; the sensitivity analysis with manufacturer-level cluster-robust standard errors accounts for this correlation in the regression estimates, but the descriptive counts and proportions remain authorization-based rather than product- or manufacturer-based. Seventh, this study did not evaluate the quality of the clinical evidence supporting authorization, prospective validation, transparency of reporting, model performance, or postmarket safety, dimensions on which important shortcomings have been documented for AI/ML devices [34]. Eighth, the specialties described as emerging were defined descriptively from Era 3-to-Era 4 growth in mean annual authorization rate; none showed a statistically significant Cochran-Armitage trend after Bonferroni correction across the 17 advisory committee panels (all Bonferroni-adjusted *P*≥.33), and the rate ratios for specialties with very small Era 3 counts are unstable. These increases should therefore be regarded as recent, era-specific observations requiring confirmation in subsequent authorization years rather than as established trends.

### Conclusions

This study documents that the clinical specialty distribution of FDA-authorized AI/ML medical devices showed measurable, era-dependent diversification during 2023 to 2025; whether this diversification represents the beginning of a sustained trend will require observation beyond this study period. Radiology remains the dominant specialty by a wide margin, accounting for 77.5% (614/792) of Era 4 authorizations; nevertheless, the radiology share peaked in Era 3 and declined significantly in Era 4, while neurology, anesthesiology, pathology, and gastroenterology-urology showed the largest Era 3-to-Era 4 increases in mean annual authorization rate among nonradiology specialties, none of which reached significance as trends across all 4 eras after correction for multiple comparisons (all Bonferroni-adjusted *P*≥.33), and cardiovascular devices remained the largest nonradiology category in absolute terms while declining in relative share. Diversification was associated with start-up and technology-company activity, although the technology-company estimate rests on 13 devices from 5 firms and is correspondingly imprecise; the progressive maturation of clinical data infrastructure beyond imaging is a plausible contributing condition that this study did not measure. For health systems, regulators, and innovators, these findings underscore the need to prepare for AI/ML medical device deployment across the full breadth of clinical medicine rather than within the radiology department alone.

## Supplementary material

10.2196/103040 Multimedia Appendix 1 Supplementary Tables S1 through S8 presenting detailed data and analyses supporting the main study findings.

## Abbreviations

CT: computed tomography
ECG: electrocardiogram
FDA: US Food and Drug Administration
HHI: Herfindahl-Hirschman Index
ML: machine learning
MRI: magnetic resonance imaging
OR: odds ratio
PCCP: Predetermined Change Control Plan
PMA: Premarket Approval
SaMD: Software as a Medical Device
SME: small-to-mid-sized enterprise
