# Supplementary material for: Clinical Specialty Expansion of AI-Enabled and Machine Learning–Enabled Medical Devices Authorized by the US Food and Drug Administration From 1995 to 2025: Longitudinal Content Analysis
Source: J Med Internet Res. 2026 Aug 25;28:e103040. doi: 10.2196/103040 (PMC13505343; doi:10.2196/103040)
Supplement: Supplementary file 1 [file jmir-v28-e103040-s001.docx]

**Multimedia Appendix 1. Supplementary Tables**

**Table S1.** Imaging modality distribution within radiology-panel devices (n=1,094), by era. Modality was identifiable for 629 devices (57.5%) from the device name or product code.

| **Imaging modality** | **Era 1** | **Era 2** | **Era 3** | **Era 4** | **Total** |
| --- | --- | --- | --- | --- | --- |
| CT | 2 | 21 | 74 | 103 | 200 |
| Ultrasound | 1 | 7 | 32 | 84 | 124 |
| MRI | 1 | 5 | 41 | 62 | 109 |
| X-ray/Fluoroscopy | 0 | 5 | 22 | 24 | 51 |
| Mammography | 0 | 6 | 18 | 20 | 44 |
| Dental imaging | 4 | 2 | 8 | 27 | 41 |
| Radiation therapy planning | 0 | 3 | 15 | 16 | 34 |
| Nuclear (PET/CT/SPECT) | 0 | 1 | 10 | 15 | 26 |
| General radiology / unspecified | 7 | 68 | 127 | 263 | 465 |
| Total | 15 | 118 | 347 | 614 | 1094 |

*Era 1 to Era 4: device counts. Devices for which a single modality could not be assigned are listed as general/unspecified.*

**Table S2.** Complete distribution of authorizations across all 17 FDA advisory committee specialty panels, by era.

| **Specialty** | **Era 1** | **Era 2** | **Era 3** | **Era 4** | **Total** |
| --- | --- | --- | --- | --- | --- |
| Radiology | 15 | 118 | 347 | 614 | 1094 |
| Cardiovascular | 4 | 36 | 34 | 62 | 136 |
| Neurology | 2 | 12 | 6 | 45 | 65 |
| Anesthesiology | 3 | 2 | 3 | 18 | 26 |
| Gastroenterology-Urology | 0 | 1 | 4 | 19 | 24 |
| Hematology | 6 | 4 | 5 | 5 | 20 |
| Ophthalmic | 0 | 2 | 4 | 4 | 10 |
| Clinical Chemistry | 1 | 5 | 1 | 2 | 9 |
| Pathology | 2 | 0 | 1 | 6 | 9 |
| General and Plastic Surgery | 2 | 1 | 0 | 4 | 7 |
| Microbiology | 2 | 2 | 1 | 1 | 6 |
| Dental | 0 | 2 | 0 | 3 | 5 |
| Clinical Toxicology | 4 | 1 | 0 | 0 | 5 |
| Orthopedic | 0 | 0 | 0 | 5 | 5 |
| General Hospital | 0 | 2 | 0 | 2 | 4 |
| Obstetrics and Gynecology | 1 | 1 | 0 | 2 | 4 |
| Immunology | 0 | 1 | 0 | 0 | 1 |
| Total | 42 | 190 | 406 | 792 | 1430 |

*Era 1 to Era 4: device counts. This table provides the full breakdown collapsed into the Other category in Table 1 of the main text.*

**Table S3.** Multivariable logistic regression for non-radiology authorization, including era × manufacturer interaction terms (reference: Era 1; incumbent).

| **Predictor** | **Odds ratio (95% CI)** | ***P* value** |
| --- | --- | --- |
| Era 2 (vs Era 1) | 0.43 (0.058–3.186) | .408 |
| Era 3 (vs Era 1) | 0.04 (0.005–0.380) | .004 |
| Era 4 (vs Era 1) | 0.30 (0.057–1.594) | .158 |
| SME (vs incumbent) | 6.00 (0.953–37.764) | .056 |
| Startup (vs incumbent) | 16.50 (1.832–148.606) | .012 |
| Tech company (vs incumbent) | NE | NE |
| Era 2 × SME | 1.08 (0.104–11.150) | .950 |
| Era 3 × SME | 3.00 (0.251–35.812) | .385 |
| Era 4 × SME | 0.33 (0.040–2.778) | .309 |
| Era 2 × Startup | 0.29 (0.023–3.599) | .333 |
| Era 3 × Startup | 0.98 (0.071–13.496) | .988 |
| Era 4 × Startup | 0.23 (0.024–2.184) | .200 |
| Era 2 × Tech company | NE | NE |
| Era 3 × Tech company | NE | NE |
| Era 4 × Tech company | NE | NE |

*Adding the interaction terms did not improve model fit over the main-effects model reported in the main text (likelihood ratio test χ²8=11.16, P=.19). NE: not estimable owing to quasi-complete separation in the technology-company strata (n=13).*

**Table S4.** Cochran-Armitage trend tests for authorization share across the four eras, for all 17 FDA advisory committee specialty panels, with Bonferroni correction (m=17).

| **Specialty** | **Era 1** | **Era 2** | **Era 3** | **Era 4** | **Z** | **P value** | **P (Bonferroni)** |
| --- | --- | --- | --- | --- | --- | --- | --- |
| Radiology | 15 | 118 | 347 | 614 | +5.30 | <.001 | <.001 |
| Cardiovascular | 4 | 36 | 34 | 62 | -3.44 | <.001 | .010 |
| Neurology | 2 | 12 | 6 | 45 | +0.84 | .40 | >.99 |
| Anesthesiology | 3 | 2 | 3 | 18 | +0.14 | .89 | >.99 |
| Gastroenterology-Urology | 0 | 1 | 4 | 19 | +2.34 | .02 | .33 |
| Hematology | 6 | 4 | 5 | 5 | -5.01 | <.001 | <.001 |
| Ophthalmic | 0 | 2 | 4 | 4 | -0.63 | .53 | >.99 |
| Clinical Chemistry | 1 | 5 | 1 | 2 | -3.37 | <.001 | .013 |
| Pathology | 2 | 0 | 1 | 6 | -0.51 | .61 | >.99 |
| General and Plastic Surgery | 2 | 1 | 0 | 4 | -1.63 | .10 | >.99 |
| Microbiology | 2 | 2 | 1 | 1 | -3.58 | <.001 | .006 |
| Dental | 0 | 2 | 0 | 3 | -0.44 | .66 | >.99 |
| Clinical Toxicology | 4 | 1 | 0 | 0 | -5.90 | <.001 | <.001 |
| Orthopedic | 0 | 0 | 0 | 5 | +1.74 | .08 | >.99 |
| General Hospital | 0 | 2 | 0 | 2 | -0.88 | .38 | >.99 |
| Obstetrics and Gynecology | 1 | 1 | 0 | 2 | -1.49 | .14 | >.99 |
| Immunology | 0 | 1 | 0 | 0 | -1.66 | .10 | >.99 |

*Z: Cochran-Armitage trend statistic; a positive value indicates an increasing share and a negative value a decreasing share across eras. All 17 panels were tested and the Bonferroni correction is applied across this full family of 17 comparisons; the previous version of this table reported 10 panels with a correction for m=10. The 10 non-radiology specialties listed in Table 2 of the main text are all included here. After correction, the only significant trends are the increasing share of radiology and the declining shares of cardiovascular, hematology, clinical chemistry, microbiology, and clinical toxicology devices; gastroenterology-urology is significant before correction only (uncorrected P=.02, Bonferroni-adjusted P=.33).*

**Table S5.** Herfindahl-Hirschman Index (HHI) of specialty concentration per era, with 95% confidence intervals from 2,000 bootstrap resamples.

| **Era** | **Devices (n)** | **Active specialties (n)** | **HHI** | **95% CI** |
| --- | --- | --- | --- | --- |
| Era 1 | 42 | 11 | 0.181 | 0.1338–0.3061 |
| Era 2 | 190 | 15 | 0.427 | 0.3591–0.5062 |
| Era 3 | 406 | 10 | 0.738 | 0.6841–0.7914 |
| Era 4 | 792 | 15 | 0.612 | 0.5689–0.6557 |

*HHI ranges from 0 to 1; higher values indicate greater specialty concentration. A decrease indicates diversification.*

**Table S6.** Manufacturer type classification criteria and resulting counts across 576 unique manufacturers. Criteria were applied hierarchically in the order technology company, large incumbent, startup, and SME, with each company assigned to the first category whose criteria it met; the categories are therefore mutually exclusive.

| **Manufacturer type** | **Classification criterion** | **Companies (n)** | **Devices (n)** |
| --- | --- | --- | --- |
| Incumbent | >1,000 employees and established before 2000 | 70 | 385 |
| SME | 11–999 employees | 27 | 104 |
| Startup | Founded in 2010 or later; pre-IPO or early stage | 474 | 928 |
| Tech company | Established software or consumer-technology firm diversifying into medical AI | 5 | 13 |

*IPO: initial public offering. The highest-volume manufacturers were verified individually against Crunchbase, PitchBook, and company websites; lower-volume single-device companies were classified using founding-year and naming conventions.*

**Table S7.** Sensitivity analyses for the multivariable logistic regression of non-radiology (versus radiology) authorization: odds ratios (95% CIs) for manufacturer type, by model specification.

| **Model** | **SME** | **Startup** | **Technology company** |
| --- | --- | --- | --- |
| Primary model (maximum likelihood, era-adjusted) | 4.55 (2.48–8.34) | 5.09 (3.33–7.79) | 50.62 (12.90–198.64) |
| Cluster-robust SEs at the manufacturer level (576 clusters) | 4.55 (1.24–16.68) | 5.09 (2.04–12.71) | 50.62 (7.92–323.56) |
| Authorization year as a continuous covariate (in place of era) | 5.24 (2.92–9.43) | 5.37 (3.53–8.18) | 45.28 (11.69–175.36) |
| Firth penalized logistic regression | 4.50 (2.46–8.21) | 4.99 (3.27–7.61) | 44.44 (11.71–168.65) |

Reference category: large incumbent. All P<.001 except SME in the cluster-robust model (P=.02). Point estimates in the cluster-robust model are identical to the primary model because only the standard errors change. CI: confidence interval; SE: standard error; SME: small-to-mid-sized enterprise.

**Table S8.** Verification of the specialty-by-era association under sparse-cell conditions.

| **Test** | **Statistic** | **df** | **P value** |
| --- | --- | --- | --- |
| Pearson chi-square (asymptotic) | 328.0 | 48 | <.001 |
| Monte Carlo permutation test (10,000 replicates) | — | — | <.001 |

In the 17-panel by 4-era contingency table, 51 of 68 cells (75%) had expected counts below 5. The permutation test permuted specialty labels across eras; none of the 10,000 permuted tables produced a chi-square statistic as large as the observed value. df: degrees of freedom.
